# Editorial: Fish Welfare in Aquaculture and Research—Where Are We Going?

**DOI:** 10.3390/ani15162367

**Published:** 2025-08-12

**Authors:** Constanze Pietsch

**Affiliations:** ProFishCare GmbH, Im Bösch 43, 6331 Huenenberg, Switzerland; pietsch.constanze@gmail.com

## 1. Introduction

Rapid technical developments are taking place in aquaculture, and its production is increasing globally every year by more than 4% [1]. Research is the necessary foundation for politics to define reasonable regulations and recommend guidelines for aquaculture, but also the basis for a well-functioning and sustainable industry with capable producers, state-of-the-art infrastructure and contented fish. This includes a clear definition of the husbandry conditions that should be recommended for each fish species.

All fish species differ in their life cycle, behaviour, environmental and nutritional requirements, and many more ways. This makes generalization of welfare assessment methods very difficult. In addition to this, our ability to empathize with fish and to understand their needs is limited. For a long time, even nociception in fish has been rejected [2]. Nowadays, we have been slowly trying to understand fish and their needs. However, actual fish welfare assessments are often insufficient to prevent harm from farmed fish.

Diseases and stress are the major threats for fish well-being since they affect behaviour, feed intake, and, subsequently, the growth of the fish [3,4]. Stress in farmed fish is commonly caused by handling, unsuitable stocking densities or feeding regimes, and many more reasons [5,6,7,8]. Furthermore, aggressive behaviour or handling of fish can lead to injuries, higher stress levels, and lower feeding efficiency [9,10]. In addition, it is necessary to check the hierarchies in the fish populations, since aggressive behaviour in most, but not all, fish species can be avoided by maintaining a homogenous size of the fish in a group [11,12,13]. But also sorting the fish according to their sizes too often should be avoided since new hierarchies must be established after disturbances [14]. Another procedure that cannot be avoided and commonly causes stress for fish is transport, since it includes handling, confinement, and, often, low water quality [15,16]. Finally, farmed fish can experience considerable stress at harvest and slaughter which affect not only the well-being of the animals, but also flesh quality [17].

Consequently, welfare is often defined by the absence of negative stress [18]. Good husbandry can, thus, be assumed when the behavioural and physiological abilities of the fish to cope with possible negative stressors are not overused. However, the actual version of welfare definition also considers that animals desire positive welfare states which agree with the concept of satisficing [19]. This concept assumes that animals do not try to achieve an absolute maximum welfare status for themselves and all group members, but a “good enough” well-being that can realistically be achieved under the given circumstances. Positive welfare has, for example, been achieved by structural enrichment [20]. However, the problem for welfare assessments is the complexity of factors that influence the well-being of an individual which represents an enormous task in terms of revealing clear, causal relationships.

Since nature is never free of stress, organisms have evolved to deal with specific types and degrees of disturbances. If changes in the environment are perceived, they might act as stressors that activate stress responses in animals. These responses comprise a set of neuronal, hormonal, behavioural, and physiological processes aiming at restoring biological equilibrium. Coping with stress is considered costly and these costs are referred to as the allostatic load [21]. The early stress research established the concept of the general adaptive syndrome [22] which assumed that the stress responses are rather similar in all species and for all stress scenarios. However, research in recent years has shown that this is not true. Environmental factors such as temperature and nutrition, but also social interactions, the ontogenetic stage, as well as the nature of the stressor (duration, severity, and repeated exposure) influence the extent and strength of the resulting stress responses (reviewed in Schreck et al. [23]). Hence, measuring the activation of early stress responses provides an opportunity to evaluate stress levels independently of the identity of the stressor. This is assumed to be advantageous when comparing different types of stressors (e.g., chemical stress versus social stress) or interactions of stressful stimuli. In addition, focusing on early biological responses to stimuli allows the identification of stress effects before they cause obvious and irreversible harm (e.g., tissue damage, reduced growth, or impaired health).

Early stress responses are formed in the brain. However, despite some important similarities, brain structures and functions in fish are different to mammals [24] and our knowledge on their regulations in the fish brain is still insufficient. Stress has often been investigated in fish that were subjected to pain, and it is known that the nociceptive processing takes place in several different brain parts [25]. Further downstream, the focus of stress assessment has mostly been on parts of the hypothalamic–pituitary–interrenal (HPI) axis that connects the hypothalamic nucleus preopticus and the cells in the pituitary that produce adrenocorticotropic hormone (ACTH) and melanocyte-stimulating hormones (MSH) to the head kidney, where the stress hormone cortisol is produced [26]. The HPI axis, thus, connects stress responses to immune system function and susceptibility to disease since immune cells are sensitive to cortisol [27]. A common response of fishes to stress is the active fight-or-flight reaction, but also passive freeze-hide reactions are observed depending on the species and personality of the animals [28]. However, fish exposed to the same stressors repeatedly stopped to escape eventually but still changed their swimming speed after stress exposure [29].

Earlier studies have already revealed that both acute stress and chronic stress affect circulating stress hormone and growth hormone levels in fish [30,31]. Chronic stress has also been shown to change the protein patterns in plasma [32]. The changes in gene expression in the fish brain also differ in acutely and chronically stressed fish and indicate changes in inflammatory genes as well [33,34]. However, predictability of the stressors as well as intensity of the experimental procedures also influence the stress responses in fish [35,36]. Thus, stress indicators such as cortisol levels appear to be unreliable, especially in chronic stress situations. The main reasons for this are individuals showing a high variability of the response levels, cortisol levels returning to basal levels within minutes or hours in acutely stressed fish, adaptations to stressful situations, and factors such as life stage, maturity, social interactions, the level of domestication, previous experiences, and the nutritional status affecting the cortisol secretion as well [37,38,39,40,41,42]. In addition, stress responses also appear to be influenced by the subjective and perceptual experience of the stressor by each fish allowing fish to be psychologically stressed [43].

For on-farm welfare assessments, research has often focused on stress research in fish, applying approaches that focused on tertiary stress responses, e.g., growth and vulnerability to diseases [44,45]. While these effects are absolutely relevant for fish farmers, earlier stress responses are of far more interest for efficient management and immediate assessment purposes. While influences of emotions on behaviour, neurophysiological, and cognitive components are not questioned in higher vertebrates, the action of affective states on these endpoints is less well understood in fish [46,47] or even questioned [48]. Since humans cannot feel as a fish would, it is necessary to describe the neuronal, behavioural and physiological reactions, and pathways in fish as best as possible.

Over the last few years, research evaluating the localization of fish’s distress responses in the brain has been conducted [31,49], but a rare number of studies differentiate between positive and negative stressors [34,50,51]. However, this information is assumed to be of fundamental importance if aspects of early stress responses in fish are to be used to judge their well-being under different rearing conditions to develop optimal guidelines for good on-farm fish management. What is also relevant for on-farm welfare assessments for fish is the practicability of the endpoints that are analyzed. So-called operational welfare indicators (OWIs) are fit-for-purpose [52], whereas Laboratory-based Welfare Indicators (LABWIs) for assessing welfare require lab equipment and often more time for the analyses.

Hence, this Editorial summarizes the current concepts and ways to assess welfare in commercial fish farms and reviews the commonly used categories of welfare parameters. The final goal of this review is to recommend ways to better assess fish welfare in the future.

## 2. Views on Fish Welfare

By farming, it is possible to select animals that offer better nutritional returns per unit expenditure than wild individuals, thereby increasing food supplies. In contrast, fish reared for research purposes are often used in certain experimental set-ups in which the fish batches should show low group variation to allow better statistical calculations. The experiments include a wide range of different procedures and purposes, such as toxicological tests or in vitro organ cultures, so that the requirements with respect to the fish health and performance in research differ considerably from the ones in aquaculture. Generally, fish rearing includes both responsibility during husbandry and additional obligations stemming from ownership. Furthermore, both aspects require the respectful handling of the fish and responsible management of the fish facilities. The ethical requirements for fish farming therefore comprise adequate space for the fish, responsible use of the available resources, limiting the environmental impacts of farming, and organizing sufficient training for staff in assessing and managing fish welfare. With respect to the stocking density, governments have released specific guidelines on how many fish can be reared in indoor facilities and in off-shore cages since high stocking densities often negatively affect fish welfare [53]. Large-scale fish farming also requires the use of natural resources. Another important aspect of ethical fish farming concerns the reduction in impacts on the environment. The most relevant environmental impacts comprise escaped farmed fish originating from foreign genetical stocks mating with wild individuals, the transfer of parasites and viral diseases to wild fish populations, and the input of large amounts of nutrients into natural water bodies [54].

Authorities want clear recommendations for rearing fish, while fish farmers want clear information on the monitoring that is necessary. Fish farmers also want support from researchers to develop better rearing conditions guidelines for farmed fish. Good husbandry can be assumed when the behavioural and physiological abilities of the fish to cope with possible stressors are not overused. The problem is the complexity of factors that influence the well-being of an individual fish.

### 2.1. Environmental Welfare Parameters

Parameters such as temperature, dissolved oxygen, and pH are commonly used to assess water quality in fish rearing systems. Their usefulness for scoring fish welfare in on-farm assessment studies is variable. Unfortunately, ensuring optimal ranges of environmental parameters alone does not mean that fish possess a good welfare status. Furthermore, water quality as a controllable factor is far more relevant for welfare assessments in land-based, recirculating fish facilities [55]. In contrast, off-shore facilities are exposed to environmental parameters, but the water quality and the weather conditions cannot be changed. By selecting the location of an off-shore facility, the environmental conditions are set. Nevertheless, sub-optimal environmental conditions in off-shore facilities contribute to the health impairment of the fish, especially if the conditions remain unsuitable for longer time periods, which should be the reason for including these parameters in welfare assessments for off-shore facilities as well. Fortunately, increased digitalization in fish farms will yield more detailed data sets containing environmental parameters that can be used for improved welfare assessments.

### 2.2. Fish-Based Parameters

Fish-based welfare parameters can be categorized as invasive, minimally invasive, and non-invasive according to the physical contact to the fish that are needed. For the assessment of fin condition or wounds, minimally invasive or non-invasive assessments are possible. Furthermore, fin condition and lesions have been recommended for monitoring and calculating welfare indices [56,57]. The reason for this is the fact that fin damage is more than an esthetic issue for farmed fish and can be used as a fish-based welfare indicator. Impaired fin condition indicates erosion due to bad water quality, mechanical abrasion when unsuitable materials have been used for the construction of the rearing facilities, infections, or aggressive behaviour of conspecifics [58]. Severe damage to fins can also allow pathogens to enter the body leading to infections causing loss from decreased growth performance, increased cost of disease treatments, or even results in higher mortality. Consequently, regular fin condition monitoring can improve the welfare of farmed fish. But the question remains which fins are most relevant for the respective welfare issues, and which fin indices represent the best way to assess fin conditions for each fish species.

Nevertheless, there are a number of additional fish-based parameters that show potential for reliably indicating the well-being of fish. For example, the handbook for the welfare of salmon was published in 2018, evaluated over 90 welfare indicators, and ended up with the recommending approximately 50 welfare indicators [52], which make welfare assessment quite complicated during routine farm work. However, this handbook made the important differentiation between fit-for-purpose Operational Welfare Indicators (OWIs) and Laboratory-based Welfare Indicators (LABWIs) for assessing welfare in a range of different fish production systems. Typical OWIs at the level of individual fish include skin condition, gill health, or the presence of parasites [59]. In contrast, group-based factors like behaviour or appetite can be used as OWIs in fish welfare assessments.

The most famous LABWI is probably the measurement of stress hormone levels. Similarly to higher vertebrates, fish produce stress hormones as a response to the exposure to stressful situations. Hence, methods to monitor cortisol levels in blood, body homogenates, scales, or water have been developed [60]. Furthermore, there are several options to measure the main stress hormone, cortisol, in fish [60]. The most common detection method for cortisol in fish are enzyme-linked immunosorbent assay (ELISA) techniques or radio-immuno assays, which are not methods that can be routinely analyzed directly on-farm. For a long time, it has been assumed that stress hormone levels are sufficient indicators of the stress levels in fish. This may be true if fish such as salmon are acutely stressed [61]. However, the study by Madaro et al. [35] indicated that heavily stressed fish do not show increased blood stress hormone levels, and the study of Opinion et al. [62] even demonstrated a down-regulation of the stress hormone levels in Atlantic salmon after exposure to long-term stress. In addition, further studies demonstrated that stress hormone levels as well as plasma glucose and lactate levels may not be optimal for stress assessments in fish farms [32,63].

It should also be noted that many fish-based parameters, such as growth and feed uptake, are assessed at the population level and not for individual fish. However, welfare at the population level should not assume homogeneity of the welfare of each group member, since it cannot be expected that all individuals in a group are similar in terms of state, sensitivity, and perception of their well-being at any point in time [19]. Vice versa, it is not possible to estimate the welfare at the population level based only on the assessments of individual welfare states, since the welfare status of each individual may vary over a considerable range depending on the stress-coping style and the different perception of the welfare optimum. In addition, Marques Maia [64] proposed that it is of special importance to investigate the individual variation in welfare in a group to improve our understanding of the welfare of fish populations, especially if the investigations take place in real farm settings. Consequently, the welfare assessments should consider both individual and population-level welfare indicators to respect both levels of the well-being of animals living in groups. However, it is also possible that individual and population welfare are in conflict, or it is impossible to accurately assess the welfare status of individuals in very large groups. In these situations, facility managers often consider maximizing the population’s welfare at the expense of individuals [65]. In future, it would be recommended to use ethical decision-making regarding animal welfare issues that clearly communicates possible concerns and helps to find a collaborative way to respect welfare states of both individuals and the entire population.

Although, it is an invasive method so far, it is suggested that the best way to identify stress in fish is achieved by investigating early responses to these stressors via the evaluation of gene expression patterns in different regions of fish brains [31,50]. To measure regulations at the brain level, PCR methods are often used. But the identification of suitable bioindicator genes for early stress response in the brain in the different fish species used in aquaculture is complicated and different stress scenarios will have to be applied to develop robust markers for stress. At the moment, stress is often evoked by air exposure, chasing, and confinement [31,51]; other husbandry problems, such as the effects of different stocking densities, the lack of knowledge on environmental enrichment in fish tanks, the perception and response to natural and anthropogenic chemicals despite the known biochemical detoxification pathways, and the emotional status of fish before, during, and after an infection with pathogens, should be addressed by researchers. A reliable method for stress assessment in fish would allow a unique description of optimal rearing conditions for fish in aquaculture and in research. This would result in the possibility to take early actions in order to resolve any rearing problems, making remediation more effective and prevent long-term harm to fish in the culture.

### 2.3. Management and Experience with Fish

Another aspect that should be considered when selecting welfare parameters is the reliability of the measured features. In contrast to environmental-based welfare indicators, the animal-based parameters are more susceptible to inter-observer variation during the assessment of these features [66], especially if these concern the appearance or the behaviour of the individuals. His may result in less reliable welfare assessments. However, the more people work with fish, the better they can judge the welfare of fish. In addition, accidents leading to poor fish welfare or even death can be avoided. Furthermore, well-trained personnel can use proper hygiene measures. For example, a quarantine for in-coming fish is a useful measure for preventing disease outbreaks and stress for the fish [67].

### 2.4. Available Tools for Fish Welfare Monitoring

Aquaculture production is increasing but the guidelines that guarantee fish welfare are lacking for many fish species. Although technical progress has been made in this field, our understanding of the biology of fish themselves is far less developed, and unfortunately, not enough attention has been paid to the well-being of fish. Well-being of fish is assumed when fish grow and show no signs of diseases. However, suitable, reliable, and accurate indicators of well-being are lacking.

Simple models for fish welfare assessment often lack the integration of a number of fish-based parameters. The welfare models SWIM 1.0 [68] and SWIM 2.0 [56] have been developed and used for on-farm welfare assessment of salmon [69]. Furthermore, detailed handbooks for the welfare of salmon and trout, evaluating more than 90 different welfare parameters, have been published [52,70]. The individual welfare parameters that have been selected for these assessments, e.g., eye condition or gill scoring, reflect distinct and very basic welfare features of the fish, such as feeding or respiration [56]. Welfare, according to these scoring systems, has mostly been evaluated for Atlantic salmon, lumpfish, and tilapia [69,71,72], but also for sea bream and sea bass [57,59]. Four scores are commonly used [71,72,73], but three scoring levels for each parameter have been defined [74], which makes comparison of the different studies complicated. Still, the most important problems in aquaculture at the moment are related to husbandry conditions that cannot be correlated to the well-being of fish because the accurate bioindicators for stress assessments are lacking. Nevertheless, the fish welfare assessment for tilapia based on a broad variety of nutritional, environmental, and health parameters showed differences for fish reared in three different semi-intensive farms [73]. But in comparison to other welfare assessments, it is obvious that certain welfare indicators appear to be more suitable to indicate a poor welfare status than others.

One indicator that naturally changes over time is the body condition, which is calculated from the body length and body weight [75]. Since the increase in length and the weight gain of growing fish does not necessarily have to be isometric [76], changes in body condition can be expected. Nevertheless, stress and diseases are known to change the appetite and feed ingestion in fish, with subsequent consequences for the weight gain of the animals [77,78,79,80]. Hence, changes in body condition show normal changes over time, but can also indicate problems with the well-being of the fish especially when they do not show increases in weight within certain periods of observation.

Other rearing conditions are clearly related to the well-being of fish at certain life stages. One example is the effect of stocking density on trout welfare [81,82], but these results have not been integrated into a welfare index calculation in that study. Moreover, interspecific differences in the preferred stocking densities exist, making general recommendations more difficult [82,83,84].

Another issue is the lack of knowledge on the effects of environmental enrichment on the well-being of fish, although a number of fish-based parameters can be positively influenced, for example, by structural enrichment [85]. However, environmental enrichments may include physical as well as sensorial cues and/or social interactions, and the response of fish to these stimuli is mostly determined by the predictability and variability of the cues [86]. Accordingly, improved growth performance and reduced aggression in Nile tilapia (*Oreochromis niloticus*) has been achieved by tactile body stimulation [87]. However, species-specific differences in the effects of environmental enrichment exist [88] and many studies did not find significant environmental enrichment effects [89]. In contrast to the obvious positive effects of physical enrichment, unsuitable materials and cracks or holes in the structures may lead to fin or skin damage, increasing the risk of infection, stress, and mortality (e.g., [90,91]). In addition, some structures or objects may favour the accumulation of organic particles and parasites, compromising health and the overall fish welfare [88]. Furthermore, structures in rearing tanks that are made of plastics may also leak out microparticles and potentially hazardous chemicals to the environment [92,93].

Moreover, natural toxins may affect the well-being of farmed fish. One example are the effects of feed-borne natural toxins as a result of using land-based crops for fish feeding; they certainly affect the well-being of farmed fish, since it has been shown that there is a correlation between dietary toxin uptake and the growth performance of fish [94]. Another example would be the disease treatment with potentially toxic chemicals that require repair mechanisms in the fish and, therefore, energy investment by the fish that must be allocated to such pathways in the fish body [95] and, thus, cannot be used for other body functions anymore. Finally, our increasing contamination of the aquatic environment with foreign chemicals will also lead to increasing effects of these anthropogenic chemicals on the fish physiology. In some cases, the effects of these chemicals on stress perception and fish behaviour have been observed [96,97].

Non-invasive welfare assessment tools are desired for good reasons. A study of the skin mucus proteomics of gilthead sea bream, *Sparus aurata* [98], identified a number of interesting proteins for assessing fish welfare, but be these will have to be validated by future studies with one or more varying rearing conditions for the fish, including different environmental, nutritional, or pathological parameters.

## 3. Welfare of Fish in Aquaculture

### 3.1. What Is Important for Fish Farmers and Facility Managers?

Certain functional traits of fish are desired in aquaculture [99], which include phenological, behavioural, morphological, as well as a number of physiological fish characteristics. Selective breeding is, therefore, commonly used to optimize the performance of the fish in aquaculture. However, external influences affect selective breeding efforts, including the need for higher feed, water and energy efficiency, and increasing sustainability [100,101]. Therefore, it is not surprising that aquaculture optimization concerns adaptations in nutrition, genetics, and the management of farms [102]. The connection of adequate fish welfare and a higher growth performance, less disease susceptibility, and lower mortality is evident. In contrast, if fish are already in a poor condition, negative influences and responses to them occur that are difficult to reverse. This is why further research is critically needed and will revolutionize the recommendations for fish welfare.

If the efficiency of animal production should be assessed, the parameter that is most frequently calculated is the feed conversion ratio (FCR). The FCR is calculated as the ratio of the amount of feed administered over a certain period and the weight-gain achieved by this. The FCR values for different fish species can vary considerably [103], depending on their rearing conditions, health status, and feed quality. Higher inclusion rates of plant-based proteins into fish feeds to increase the sustainability of aquaculture production often increases FCR values. Since there is normally a species-specific range for the FCR of a certain fish species, for economically important species, genetical improvements have been proposed to further optimize the feed efficiency and growth performance while limiting the environmental impacts at the same time [104].

### 3.2. What Are the Main Issues in Aquaculture?

Different rearing systems are used for fish rearing in aquaculture, including extensive and intensive rearing systems, flow through and recirculating systems, and off-shore cages and ponds. Furthermore, over 200 different fish species are currently used in aquaculture [1], which makes generalizing welfare guidelines difficult. The main problems in aquaculture are malnutrition, cannibalism, deformations, diseases, environmental pollution, potential damages of fish due to sorting and handling, and stress deriving from all these aspects [105,106]. Diseases in particular are an important threat for fish welfare [107]. Therefore, many welfare assessment methods include this aspect for welfare index calculation [56,68,108] and entire books for assessing the welfare of aquaculture-relevant fish species have been published [109].

In addition, proper rearing conditions are especially important for juveniles for their later performance, especially if technopathies, epigenetic effects, effects of transport, and handling are taken into account [110,111,112]. But the requirements of farmed fish are highly species-specific. This is, for example, true for the parameters relevant for water quality: temperature, pH, dissolved oxygen, CO_2_, and conductivity. Another aspect that is often addressed separately for each fish species is their nutrition. Hundreds of fish species are reared in aquaculture, making it difficult to know the nutritional needs of all fish species at all life stages. However, a lot of effort has been invested to describe the needs of commonly farmed fish in order to produce fish feed that meets the requirements of the different fish species and different life stages [113,114]. Fish feed formulation must consider the different dietary requirements of farmed fish [115]. Specific diets have not been developed for all species in aquaculture. In addition, the development of sustainable aquafeeds for the future is a substantial challenge [116]. But fish nutrition is not only important to ensure the optimal growth of the animals. The intestinal microbiome and effects on welfare and the behaviour of fish has been investigated [117,118]. Finally, domestication profoundly determines how fish cope with the artificial rearing conditions in aquaculture [119]. Hence, fish show adaptations to their rearing conditions [120], but controlled breeding programmes supported by genomic analyses have not been applied to many fish species so far [121].

### 3.3. Benefit of Welfare for Aquaculture

Fish farmers desire better growth performance and better fish health, leading to higher profits. Accordingly, a survey revealed that fish farmers expect improved product quality and higher farm productivity when considering the welfare of their fish [122]. In addition, following welfare guidelines is also expected to positively affect the reputation of aquaculture and selling opportunities.

## 4. Fish Welfare in Science

### 4.1. Which Fish Species Are Used?

More than 80 different fish species are used for research purposes in Europe, North America, and Oceania, with zebrafish (*Danio rerio*), rainbow trout (*Oncorhynchus mykiss*), Atlantic salmon (*Salmo salar*), goldfish (*Carassius auratus*), medaka (*Oryzias latipes*), and carp *(Cyprinus carpio*) being the dominant species [123].

### 4.2. What Is Important for Science?

The main goal should be a uniform fish stock used for later experiments, because variation in fish health and welfare would probably increase the response variation in the subsequent experiments. During the experiments, controlling the rearing parameters is a prerequisite to experiments that are inter-comparable with other research labs. In addition, experiments should, in the optimal case, expose the fish only to one distinct stressor or a well-described mixture of stressors and exclude the influence of other factors as much as possible to allow the description of causal relationships between the stressors and the responses in the fish.

If fish of poor health are used, the experiments will most likely produce inferior data, the studies may be not valid, and repeated testing may be required, which would increase the number of animals used. Toxicity tests are commonly performed according to regulatory guidelines, which are very strict with respect to the scientific validity criteria. But lethal endpoints in particular lead to obvious suffering of the animals and better endpoints for lethal toxicity tests may be applied in the future [124]. Compared to the strict application of the scientific validity criteria for toxicity tests, husbandry and handling procedures are often not described in much detail in the regulatory guidelines. However, improving husbandry conditions is important and implications for improved fish welfare and study reproducibility have been noted [125]. Hence, scoring sheets for monitoring the well-being of the experimental fish have been proposed to allow better monitoring of the welfare of experimental fish [108].

Since diseases are a major cause for poor health in fish at research facilities, monitoring of the bacteria, viruses, and parasites is recommended. In addition, a number of non-infectious reasons for health impairment exist, such as insufficient rearing conditions, malformation, aggression, and abnormal behaviour. However, the survey by Mocho and von Krogh [123] showed that one quarter of the research facilities using zebrafish did not monitor the main causes of infections regularly, although relevant diseases appear to be present quite frequently.

Stocking density is also an important factor for the well-being of fish reared in research facilities. Stocking density profoundly determines individual access to feed sources, affects water quality, allows the potential transfer of parasites and infectious diseases, and interactions with conspecifics, including mating [126]. In addition, stocking densities also appear to affect the influence of structural enrichments [127].

Mortality appears to be recorded regularly in most research facilities, but the number of facilities also investigating the reasons for mortalities based on well-defined criteria was rather low [123]. Unfortunately, stress is less frequently recognized as a cause of unhealthy fish in research. The Use of Fishes in Research Committee [128] recommended minimizing the handling of fish, respecting the osmoregulatory requirements of the fish during and after the handling, changing the environmental conditions for the fish only stepwise and not rapidly, using adequate stocking densities, and giving fish time to cope with stressors. In addition, three quarters of the survey participants did monitor environmental or feed samples less than every year and 38% did not at all [123]. These numbers show that the knowledge on impaired health due to suboptimal environmental conditions, including low water exchange rates or unhealthy feed, is insufficient [123,129].

### 4.3. Benefit of Welfare for Research Outcomes

Fish with good health respond better to the treatments, so are more homogenous. Fewer fish would have to be used in experiments and better study results could be expected. Thus, more standardization of rearing conditions for fish in research is highly recommended.

## 5. Conclusions or “What About the Future”?

Fish welfare is important, regardless of the final use of the fish. In aquaculture, there is a tendency to use more fully automated monitoring systems and even artificial intelligence to improve fish farm management. For example, increasing digitalization increases the production efficiency in aquaculture with respect to time, labour, and costs [130]. Accordingly, operations in aquaculture are increasingly supported by digital techniques. For example, machine learning techniques are used in aquaculture, allowing further automation and predictions, including fish behaviour and water quality [131]. However, the supply chains in aquaculture are affected [132] by digital techniques supporting the globalization of business activities, leading to more efficiency and productivity, and simultaneously reducing waste and pollution. Thus, more digitalization is assumed to be very important for the future of aquaculture [133]. But digitalization also leads to disruption of the aquaculture industry, with a number of new technologies profoundly affecting the way fish is produced. These new digital technologies include robotics, 3D printing, drones, new types of sensors, artificial intelligence, augmented and virtual reality, cloud-edge computing, and blockchain [131,132]. For example, real-time monitoring via sensors makes the Internet of Things possible and enables autonomous fish production in aquaculture; however, infrastructure-related issues often limit its use [134]. Combining several of these technologies, including machine learning, computer vision, and the Internet of Things, results in so-called Smart Farming [135]. But the reason for the development towards Smart Farming is not only the availability of new technologies. The increasing need for more sustainability and better disease control are also the main drivers for this trend [135]. Nevertheless, the greatest challenge is currently the establishment of digital twins in aquaculture [136] that will further foster the so-called Precision Fish Farming, which is important for the future of aquaculture, since it is assumed to be a key factor for improved sustainability in production [137].

In case of sufficient data availability that allows the desired simulations, a digital twin can be established as a virtual representation of a real fish facility system, resulting in optimal management of the farm, including proper visualization, prediction, monitoring, optimization, and control of the system states, as well as improved decision-making [138]. The main advantage of digital twins is the testing of certain scenarios in the digital facility before changes are made to the actual facility, which reduces the number of fatal decisions during operations. To prepare the aquaculture sector for the digital disruption, a Hub approach has also been recommended, which can grant access to specialist equipment and expertise to deliver the required real-time solutions for the industry [132]. A Hub could also deliver adequate information to policy makers and would allow the engagement of the society by enabling increased consumer awareness and potential end-user feedback.

However, simulations and classifications as part of a data-centric approach in the aquaculture industry require sufficient amounts of data of an acceptable quality. High-quality data can be collected if the methods of data sampling, curation, and storage address the spatial and temporal complexity and heterogeneity of the data [139].

Hence, the development of methods in data science contributes to the development of digital businesses. Based on this, increasing the digitalization of fish farms unquestionably allows better farm management. Therefore, digital transformation has also changed the way aquaculture facilities are managed—leading to Aquaculture 4.0 [140]—in parallel to the transformation observed in many other industrial sectors. However, digitalization is expensive, and in most cases only large-scale farms can afford such systems. Still, global fish production must become more sustainable and respect animal welfare. Therefore, combining fish monitoring and treatment of diseases, for example, is a promising strategy.

One major advantage of artificial intelligence used in aquaculture lies in an improved management of water quality [141], but also artificial intelligence-supported predictive analytics increase sustainability by enabling the forecasting of environmental impacts, optimizing resource allocation, and reducing the generation of unnecessary waste [135].

Environmental parameters for the optimal rearing of zebrafish, sea bass, sea bream, Atlantic salmon, and common carp have been summarized by Toni et al. [142]. The clear description and recommendation of fish-based parameters has been slow. Pavlidis et al. [143] have already recommended the establishment of digital tools and standardized procedures for identifying and monitoring OWIs for farmed salmon. The use of automated parasite counting systems may also solve the problem of individual human bias, as observed in a recent study on lice infestation threshold levels in salmon, which influence sea lice counts reported by fish farmers [144]. Furthermore, if new fish species are used in research or in aquaculture, it is recommended to conduct smaller pilot studies to learn more about the stress responses of this species before more specific trials are performed.

Finally, the review by Gilmour and Bard [145] indicated that the social buffering of stress in fishes takes place, whereby the stress responses of individuals are influenced by the presence of conspecifics. Moreover, social buffering can influence hormonal, behavioural, as well as neural stress responses in fish. The resulting dampening of the HPI axis is thought to counteract the possible social transmission of stress in fishes [146,147] or enhance the capacity of individual animals to cope with stressful situations [148,149]. This further emphasizes that research must consider a high number of factors, features, and interactions in order to conduct useful studies that support the future development of welfare concepts and models for aquaculture and fish research.
